# WHEN THE GOING GETS TOUGH, GERONTOLOGY (AND OTHER HEALTH PROFESSIONS) STUDENTS GET GOING

**DOI:** 10.1093/geroni/igae098.1393

**Published:** 2024-12-31

**Authors:** José Baralt

**Affiliations:** University of Puerto Rico, San Juan, Puerto Rico

## Abstract

In September of 2017, two category 5 hurricanes hit Puerto Rico. These hurricanes caused massive destruction and close to 5,000 excess deaths. Thousands of people lost everything, and many communities were isolated for weeks. The whole island was without electric power for several days, and some areas in the mountains did not get power back for 6-8 months. Hopelessness started to take over. The bravery and drive of a community leader who came asking for our help was the spark we needed to stand up and get going. Dozens of our students delivered food, water, medications, and healthcare services to those in need for several months. In January of 2020 these students and faculty went back to work in full force after a series of devastating earthquakes shook the island. And, to this day, our team still visits some of these communities, just to check on them and refer people with significant needs to the appropriate agencies. All this without skipping a beat in our academic work. Where did we find the fortitude to keep going with our community work AND our academic work? We did have three main sources of inspiration: 1) community leaders who rose above every possible challenge; 2) the communities themselves; and 3) our students and colleagues. The key behind this amazing response was that those of us who work in service professions thrive when we are needed. And our students certainly shone in the darkest period our island has lived in for many decades.

